# Current insights in noise-induced hearing loss: a literature review of the underlying mechanism, pathophysiology, asymmetry, and management options

**DOI:** 10.1186/s40463-017-0219-x

**Published:** 2017-05-23

**Authors:** Trung N. Le, Louise V. Straatman, Jane Lea, Brian Westerberg

**Affiliations:** Division of Otolaryngology - Head & Neck Surgery, Department of Surgery, University of British Columbia, Vancouver, BC Canada

**Keywords:** Noise-induced hearing loss, Occupational hearing loss, Asymmetrical hearing loss, Sensorineural hearing loss

## Abstract

**Background:**

Noise-induced hearing loss is one of the most common forms of sensorineural hearing loss, is a major health problem, is largely preventable and is probably more widespread than revealed by conventional pure tone threshold testing. Noise-induced damage to the cochlea is traditionally considered to be associated with symmetrical mild to moderate hearing loss with associated tinnitus; however, there is a significant number of patients with asymmetrical thresholds and, depending on the exposure, severe to profound hearing loss as well.

**Main body:**

Recent epidemiology and animal studies have provided further insight into the pathophysiology, clinical findings, social and economic impacts of noise-induced hearing loss. Furthermore, it is recently shown that acoustic trauma is associated with vestibular dysfunction, with associated dizziness that is not always measurable with current techniques. Deliberation of the prevalence, treatment and prevention of noise-induced hearing loss is important and timely. Currently, prevention and protection are the first lines of defence, although promising protective effects are emerging from multiple different pharmaceutical agents, such as steroids, antioxidants and neurotrophins.

**Conclusion:**

This review provides a comprehensive update on the pathophysiology, investigations, prevalence of asymmetry, associated symptoms, and current strategies on the prevention and treatment of noise-induced hearing loss.

## Background

Exposure to excessive noise is the most common preventable cause of hearing loss. It has been suggested that 12% or more of the global population is at risk for hearing loss from noise, which equates to well over 600 million people [1]. The World Health Organization estimated that one-third of all cases of hearing loss can be attributed to noise exposure [2]. Noise-induced hearing loss (NIHL) has long been recognized as an occupational disease, amongst copper workers from hammering on metal, blacksmiths in the 18^th^ century, and shipbuilders or “boilermakers” after the Industrial Revolution [1–3].

Without doubt, chronic noise exposure and the resultant cochlear trauma cause hearing loss and tinnitus. In the United States among workers not exposed to noise, 7% have hearing loss, 5% have tinnitus, and 2% are afflicted with both hearing loss and tinnitus. However, among noise-exposed workers the prevalence is significantly higher at 23, 15 and 9%, respectively [4]. Within a group of one million noise-exposed workers, the highest risk occupations for hearing loss were identified to be those in mining, wood product manufacturing, construction of buildings, and real estate and rental leasing [5]. Hearing loss was more prevalent among men than women, likely due to a disproportionate number of males in these occupations, and the risk of hearing loss increased with age.

Despite its prevalence, there is still an ongoing debate about the consequence of the noise-induced damage. For many years, the maximum severity of NIHL was argued to be mild to moderate and symmetrical based on pure tone audiograms [6]. The impact of hearing loss might be underestimated as recent studies have shown evidence for hidden hearing loss and synaptopathy-induced poor speech recognition [7, 8]. Furthermore, the additional impact of noise-induced tinnitus and vestibular dysfunction is still not fully elucidated.

The objective of this review is to provide a comprehensive overview of NIHL including the fundamental and advanced pathophysiology, specific investigations, including detailed discussion on asymmetric NIHL, associated symptomatology, available interventions for prevention and treatment.

## Pathophysiology of NIHL

### Fundamental equal-energy principle

NIHL is a complex disease that results from the interaction of genetic and environmental factors, but is generally still dictated by the extent of biological damage caused by noise exposure. The total amount of noise to which an individual is exposed can be expressed in terms of energy level. The energy level is a function of the sound pressure of noise (in decibels) and of the duration of exposure over time. The equal-energy principle effectively states equal energy will cause equal damage (in any given individual), such that similar cochlear damage may result after exposure to a higher level of noise over a short period of time as would occur after exposure to a lower level of noise over a longer period of time [9].

### Environmental factors

For environmental exposure, hearing loss can be caused by long-term, continuous exposure to noise and is generally referred to as NIHL. However, hearing loss can also result from single or repeated sudden noise exposure, which is generally referred to as acoustic trauma. Exposure to sudden impulse noise is more detrimental than exposure to steady state noise [10]. This review is largely focussed on the former.

Noise trauma can result in two types of injury to the inner ear, depending on the intensity and duration of the exposure: either transient attenuation of hearing acuity a.k.a. temporary threshold shift (TTS), or a permanent threshold shift (PTS) [11]. Hearing generally recovers within 24–48 h after a TTS [12]. However, recent studies using a mouse model have found TTS’s at young ages accelerated age-related hearing loss, even though the hearing thresholds were completely restored shortly after the TTS [13]. Longitudinal data on the impact of TTS’s on the human ear, however, are lacking.

The recovery of TTS is probably a result of reversible uncoupling of the outer hair cell stereocilia from the tectorial membrane [14] and/or reversible central gain increase and associated hyperacusis and tinnitus [15]. However, even when there is recovery of auditory pure tone thresholds, there can be considerable damage to the ribbon synapses, a rapid degeneration termed synaptopathy [7, 8]. Synaptopathy results in loss of connections between the inner hair cells and their afferent neurons in the acute phase of noise-induced cochlear trauma [7, 16], and is most likely a result of glutamate excitotoxicity causing damage to the post-synaptic terminals [8]. This is also referred to as Noise-Induced Hidden Hearing Loss, as it is not accompanied by a pure-tone threshold shift [8]. Although the extent to which synaptopathy contributes to NIHL is unknown, it is argued that these synaptopathic mechanisms, similar to synaptopathic disease in certain types of auditory neuropathy, are involved in NIHL [17]. This is also supported by research in animals showing intact hair cells but extensive noise-induced spiral ganglion loss [7].

The characteristic pathological feature of NIHL with PTS is the loss of hair cells, particularly the prominent loss of outer hair cells at the basal turn, while loss of inner hair cells was limited. Degeneration of the auditory nerve followed the loss of outer hair cells in both temporal bone histopathology and in a mouse model [18]. A crucial characteristic of hair cell loss due to any cause (noise, ototoxic medications, age) is the inability of mammalian sensory cells to regenerate [19].

With sufficient intensity and duration of noise, not only the hair cells but the entire organ of Corti may be disrupted [20]. Destruction of the organ of Corti can be the result of two mechanisms: mechanical destruction by short exposure to extreme noise intensities or metabolic decompensation after noise exposure over a longer period of time [21]. Mechanical destruction is acquired by exposure to noise intensities above 130 dB sound pressure level (SPL) leading to disassociation of the organ of Corti from the basilar membrane, disruption of cell junctions, and mixing of endolymph and perilymph [22]. The pathology observed as a result of metabolic decompensation includes stereocilia disruption, swollen nuclei, swollen mitochondria, cytoplasmic vesiculation, and vacuolization [23, 24]. Current theories of metabolic damage center on the formation of free radicals or reactive oxygen species (ROS) and glutamate excitotoxicity evoked by excessive noise stimulation, followed by activation of signalling pathways leading to cell death [25]. ROS emerge immediately after noise exposure and persist for 7–10 days thereafter, spreading apically from the basal end of the organ of Corti, thus widening the area of necrosis and apoptosis [26, 27]. Glutamate is the excitatory neurotransmitter that acts at the synapses of the inner hair cells with the eighth cranial nerve. High levels of glutamate can over-stimulate postsynaptic cells and cause swelling of cell bodies and dendrites [28], a process referred to as glutamate excitotoxicity.

Another consequence of noise exposure is an increase of free calcium (Ca^2+^) in outer hair cells immediately after acoustic overstimulation contributed to by both entry through ion channels and liberation from intracellular stores [29]. Ca^2+^ overload can also trigger apoptotic and necrotic cell death pathways independent of ROS formation [30].

Aside from direct effects on the auditory system, noise also can cause psychological and physiological stress. The hypothalamus-pituitary-adrenal (HPA) axis can modulate the sensitivity of the auditory system and be activated by acoustic stress [31]. Mice lacking corticotropin-releasing factor receptor (a critical factor in HPA function) in the cochlea exhibited loss of homeostasis and protection against noise-induced hearing loss, leading to an increased susceptibility to noise trauma [32].

### Genetic factors

The genetic susceptibility to NIHL has been clearly demonstrated in animals. Mouse strains (C57BL/6 J) exhibiting age-related hearing loss were shown to be more susceptible to noise than other strains [33–35]. Also, several heterozygous and homozygous knockout mice including *Cdh*23 [36], *Pmca*2 [37], *Sod*1 [38], *Gpx*1 [39], *Trpv*4 [40], *Vasp* [41], and *Hsf*1 [42] were shown to be more sensitive to noise than their wild-type littermates. These studies on knockout mice indicate that there are some genetic deficits that disrupt specific pathways and structures within the cochlea and predispose the inner ear to NIHL.

The discovery of human genetic factors predisposing individuals to NIHL has been hindered by many difficulties. To date, no heritability studies have been performed, since families where all subjects are exposed to identical noise conditions are almost impossible to collect. Hence, another approach involving screening of Single Nucleotide Polymorphisms (SNPs) of different genes known to play a functional and morphological role in the inner ear has been adopted. SNPs are common point mutations in the genome (occurring every 100 – 300 base pairs), and their genotyping is believed to be a successful tool in analyzing the genetic background of complex diseases, such as NIHL. In such studies, a disease susceptibility allele is expected to occur more often among susceptible groups than resistant ones. The most promising results were obtained for the inner ear potassium (K+) ion recycling and heat shock protein (HSP) genes. K+ recycling genes are indispensable for the process of hearing, as evidenced by the fact that multiple mutations in these genes (GJB2, GJB3, GJB6, KCNE1, KCNQ1 and KCNQ4) lead to both syndromic and non-syndromic forms of hearing loss [43–46]. HSPs form a group of conserved proteins assisting in synthesis, folding, assembly and intracellular transport of many other proteins. HSPs are ubiquitously expressed in cells under physiological and pathological conditions, and their expression increases under stressful conditions, including noise exposure. When first induced by exposure to moderate sound levels, they can protect the ear from excessive noise exposure [47–50]. Three genes are responsible for HSPs synthesis: HSP70-1, HSP70-2 and HSP70-hom. Variations in HSP70-1, HSP70-2 and HSP70-hom genes were shown to be associated with susceptibility to NIHL and these results were replicated in three independent populations, Chinese, Swedish and Polish [51, 52]. Recently, the significance of genetic variation in NIHL development has also been shown for otocadherin 15 and myosin 14 genes [53].

## Audiometric investigations

### Pure tone audiogram

Early or moderately advanced NIHL usually results in the typical ‘boilermakers’ notch at 4 kHz, with spread to the neighbouring frequencies of 3 kHz and 6 kHz [54] and some hearing recovery at 8 kHz [6, 55]. The fact that frequencies around 4 kHz are most affected by noise is most likely due to the resonance frequency of the outer ear/ear canal as well as mechanical properties of the middle ear [56]. High frequencies are also typically affected by presbycusis; therefore the notch may disappear with aging, making it difficult to differentiate NIHL from presbycusis. Whether or not chronic noise exposure can also result in hearing loss at 8 kHz is debated [57]. With further noise exposure, the notch can get deeper and wider eventually involving lower frequencies such as 2 kHz, 1 kHz and 0.5 kHz [58, 59].

Hearing loss induced by noise exposure is quoted to be on average no greater than 75 dB in the high frequencies and no greater than 40 dB in the lower frequencies [6]. However, chronic noise exposure can in some individuals cause severe to profound sensorineural hearing loss (SNHL). When individual data is reviewed, severe to profound SNHL after noise exposure is documented in noise-exposed individuals with a prevalence varying from 1 to 15% [60–64], well above the prevalence among the general population in the United States (0.5%) and United Kingdom (0.7%) [65, 66]. The wide range in prevalence of severe to profound hearing loss found in studies of noise exposed populations may be influenced by underlying genetic factors, or differences in the intensity, type and duration of noise exposure. For instance, SNHL can progress to severe or profound with prolonged durations of noise exposure [67, 68], especially in impact noise [69].

### Speech recognition

Traditionally, pure tone thresholds were solely relied upon to determine the extent of NIHL, resulting in an underestimation of NIHL prevalence and functional impact. NIHL can be associated with a decrease in speech recognition scores in quiet as well as in background noise, even in the setting of a normal pure tone audiogram [16]. This is probably related to the synaptopathic mechanisms, as discussed previously [7, 8, 16] and reduced temporal processing skills [70] as a result of noise-induced affected connections between inner hair cells and low spontaneous rate auditory nerve fibres, which are important for temporal processing [8]. In order to quantify noise-induced damage, it is recommended that speech recognition tests in quiet and in noise should be performed in addition to pure tone thresholds [7].

### Otoacoustic emissions (OAEs)

Otoacoustic emissions have the necessary features to serve as an objective, sensitive, and easy-to-administer tool for the diagnosis of NIHL. In laboratory animals exposed to high noise levels, OAE amplitude reductions showed a good correlation with permanent threshold shift of more than 25 to 35 dB SPL measured by auditory evoked potentials and significant outer hair cell loss measured by histologic cochleograms [71]. Parallel decreases in pure-tone sensitivity and OAE amplitudes were reported among noise-exposed industrial workers and military personnel [72–74]. In a large sample of subjects with NIHL and normal hearing ears, the presence of click-evoked OAEs at 2 and 3 kHz could distinguish the two groups with 92.1% sensitivity (correct discrimination of NIHL) and 79% specificity (correct discrimination of normal audiogram) [75]. Similarly, distortion-product OAEs at 2, 3 and 4 kHz yielded 82% sensitivity and 92.5% specificity. Several studies have suggested that OAEs may provide an early indication of noise-induced cochlear damage before evidence for NIHL appears in standard audiometry [76, 77]. However, OAEs can only be used to monitor hearing effectively when there is room for hearing deterioration; hence, audiometry is indispensable in the presence of a pre-existing hearing loss and/or when OAEs are low or absent [78]. OAEs might be more sensitive (and perhaps very useful) with regard to detecting NIHL at an earlier, “pre-clinical” stage, although more data is needed to establish well-defined criteria for the successful use of OAEs in this clinical setting.

### Objective measures for noise-induced-synaptopathy

Electrophysiologic measurements such as ABR have been used to detect noise-induced synaptopathy [79]. There is evidence that suprathreshold wave 1 ABR responses reduced after noise exposure in animals with normal auditory thresholds, at the frequencies tonotopically related to the synaptic loss [80, 81]. Therefore it is suggested that wave 1 of the ABR can be predictive to the degree of synaptopathy [80, 81]. However, studies in human subjects have yielded conflicting results with some studies providing evidence for wave I reduction as a function of noise exposure [82], whereas others do not [83]. This variation in outcome might be caused by lack of sensitivity of ABR testing perhaps due to variations in ABR electrode placement [84], which makes the usage of wave I as a diagnostic test for cochlear synaptopathy in humans less ideal [85].

Emerging evidence suggests that acoustic reflex testing may be helpful for early detection of noise-induced synaptopathy in humans. Threshold shifts in acoustic reflexes, without audiometric hearing loss, might be caused by synaptopathy [86, 87]. Whether or not acoustic reflexes can be used to assess synaptopathy in humans requires further research.

## Asymmetric NIHL

The typical pattern of hearing loss resulting from acoustic trauma is symmetrical [6]. However, there is increasing evidence that asymmetrical hearing loss occurs as well (Table 1). Asymmetry in NIHL generates some controversy in both clinical as well as medico-legal contexts and hence warrants an in-depth discussion.

**Table 1:** Summary of literature on asymmetric NIHL

First authors & year	Design	Participants	Calculation Methods	Asymmetry Criteria	Outcomes	Additional factors considered
May et al. 1990 [101]	Case series	49 dairy farmers 94% male, 6% female mean age 43.5 average farming 29.4 years	0.5,1, 2, 3 kHz (PTA) 3, 4, 6 kHz (HFA)	>20 dB average hearing loss in either ear	Left ear is more severely affected in both groups. 37% abnormal PTA, 65% abnormal HFA. Significant association with years worked and age.	presbycusis, small sample.
Ostri et a1. 1989 [102]	Case series	95 orchestral musicians 80 males, 15 females age 22–64	0.125, 0.25, 0.5, 0.75, 1, 1.5, 2, 3, 4, 6, 8 kHz (PTA)	>20 dB average hearing loss in either ear	44% of musicians had hearing impairment attributed to occupational noise exposure. Significant poorer hearing on the left ear found at higher frequencies among violinist.	instrument played, side of orchestral band, previous noise exposure
Cox et a1 1995 [63]	Case series	235 soldiers with past weapon noise exposure age 16–55	0.5,1, 2, 3,4, 6 kHz (average single frequency threshold)	Interaural difference = asymmetry >10 dB	67% asymmetry at 4 kHz. Average hearing loss and interaural asymmetry increased with frequency.	handedness, emotional immaturity, motivation for army service, use of ear defenders
Pirila et al. 1992 [109]	Cross-sectional study	3487 random people 1640 males, 1847 females 3 age groups (5–10,15–50, >50)	0.125, 0.25,0.5,1, 2, 3, 4, 6, 8 kHz (average single frequency threshold)	Interaural difference = asymmetry >0 dB	The inferiority of hearing in the left ear at 4 kHz seems to be assiciated with noise damage. The average interaural difference at 4 kHz was more marked in age 15–50.	shooting history occupational noise exposure
Pirila et al. 1991 [98]	Cohort study	28 non-shooting normal HL 10 males, 18 females age 17–29 exposure to broad band noise 88–91 dB for maximum 8 h	4 kHz (average single frequency threshold)	determine TTS after noise exposure	TTS was greater in the left ear than the right. Negative correlation between pre-exposure threshold level.	rely on history, samll sample size.
Chung et al. 1983 [95] Audiology	Case series	1461WCB claims for NIHL no head injury, no ear surgery age 36–82	2 kHz (average single frequency threshold)	>20 dB	4.7% has asymmetry, suggesting damage toward apex. 82.6% has worse hearing thresholds in the left ear. 2 kHz is lateral difference in susceptibility to noise damage.	limited frequency considered
Nageris et al. 2007 [103]	Case series	4277 army personnel files age 16–55	3–6 kHz (PTA)	mild loss = 25–40 dB HL moderate loss = 41–60 dB HL severe loss = 61–90 dB HL asymmetry = different grade	50% symmetrical. 34.2% left asymmetrical NIHL. 16.3% right asymmetrical NIHL	No significant differences in: age, sex, type of noise, protection, length of exposure, handedness, acoustic reflex.
Simpson et al. 1993 [202]	Correctional study	1667 audiometric records of 10 industries 1367 males, 300 females mean age 32.7 and 33.5	2, 3,4 kHz (average threshold)	Interaural difference = L-R laterality >5 dB	80% unilateral with left 42% and right 38%. Baseline hearing asymmetry appears to be a precursor to unilaterality with 63% in the better ear.	no recordof otologic background, no noise exposure history.
Hong et al. 2005 [60]	Cohort study	623 operating engineers mean age 42.96 male 92%	0.5,1, 2, 3, 4, 6, 8 kHz (PTA)	Asymmetry: >15 dB at 0.5,1,2 kHz >30 dB at 3,4, 6 kHz	19% of workers had asymmetrical hearing loss. Significant poorer hearing in the left ear, especially at 4 and 6 kHz	Use of hearing protection devices resulted in better hearing but in low use
Fernandes et al. 2010 [94]	Case series	208 clients with hearing loss for compensation; age 36–73 203 males, 5 females	0.25–6 kHz (hearing threshold)	Asymmetry: >10 dB for 2 frequencies >15 dB for one frequency	22.6% of clients had asymmetrical hearing loss. Left side had greater loss in 60% of cases.	MRI showed no central pathology
Chung et al. 1983 [95] J Occu Med	Cohort study	244 shingle sawyers all males age 20–59	0.5,1, 2, 3, 4, 6, 8 kHz (average single frequency threshold)	not defined	Asymmetry of hearing loss is significant but small compared to general industrial population especially at low frequencies.	101/244 had history of shooting. Hearing protection not well-defined. Small difference of 2.8 dB to left side.
Alberti et al. 1979 [1]	Case series	1873 patients with hearing loss for WCB	0.5,1, 2, 4 kHz (PTA)	asymmetry >15 dB	15% had asymmetrical hearing loss 5.2% attributed to noise exposure	no treatable disorder found after extensive investigations.
Robinson et al. 1985 [111]	Case–control series	63 subjects with noise exposure (94 dB) of lOrs 97 normal control subjects	0.5–6 kHz (hearing threshol)	Interaural difference = L -R asymmetry >15 dB	10% left-right difference at 4 kHz.	small sample variable audiogram shapes
Berg et al. 2014 [92]	Cohort study	355 young workers age 29–33 68.5% men follow-up <16 years	0.5,1, 2, 3, 4, 6, 8 kHz (hearing threshold)	not defined	Asymmetry at >2 kHz in men Increased asymmetry with increased levels of hearing loss Asymmetry larger in men	Asymmetry varies with shooting exposure No head shadow effect on asymmetry
Dobie et al. 2014 [91]	Case–control series	1381 men with noise 80–102 dB 663 men with noise <80 dB occupational noise exposure	0.5,1, 2, 3 kHz (PTA) 3,4, and 6 kHz (PTA)	not defined	no significant asymmetry attributable to current occupational noise exposure Left ears were 1–2 dB worse than right ears for both groups	
Dufresne et al. 1988 [96]	Case series	602 WCB claims	0.25–8 kHz (hearing threshold)	not defined	more hearing loss in left ear compared to right ear (5–30 dB) in truck drivers, but not significant for others	small sample of truck drivers (n = 10)
Segal et aI. 2007 [99]	Cohort study	429 workers 241 (56.2%) with noise exposure (hearing threshold) 188 patients (43.8%) without 79.3% men with SNHL (>29 dB)	0.25–8 kHz (hearing threshold)	not defined	in noise exposed group, left ear has higher threshold in men. no significant difference left-right in group wihout noise exposure.	
Zapala et al. 2012 [203]	Case series	Case series n = 5661 benign assymmetry n = 85 vestibular schannoma	0.25–8 kHz (PTA)	asymmetry < 20 dB	Greater asymmetry in self-reported noise exposure history. Largest asymmetry at frequencies >1 kHz Asymmetry increased with age	Small differences in asymmetry: Males (5.14 dB) at 3 and 4 kHz Females (5.8 dB) at 4 kHz
Royster et al. 1980 [90]	Cohort study	industrial noise exposure 14186 (75.9% male)	0.5–6 kHz (hearing threshold)	not defined	right ears are significantly lower threshold Asymmetry is largest for frequecies >2 kHz	Mean differences in asymmetry re small (l–5 dB).
Kannan et al. 1974 [100]	Review	n = 172 50% male	l–8 kHz (mean threshold)	difference L-R >0 dB	Right ear significantly better hearing than left in males only	No data about the extend of noise exposure

*Abbreviations: HFA* high frequency average, *HTL* hearing threshold level, *kHz* kilohertz, *NIHL* noise induced hearing loss, *PTA* pure tone average, *SNHL* Sensorineural hearing loss, *STS* standard threshold shift, *TTS* temporary threshold shift, *WCB* workers’ compensation board, *dB* decibel, *L* left, *R* right

### Evidence for asymmetric NIHL

A recent systematic review concluded that the evidence for asymmetrical noise-induced trauma was limited, however only studies that reported an asymmetry of more than 15 dB were included [88]. In the general population, the incidence of interaural threshold difference of 15 dB or more is only 1% [89], whereas the incidence of asymmetrical hearing loss in noise-exposed individuals varies widely between 4.7 and 36% (Table 1). Asymmetries between left and right hearing thresholds are typically small (less than 5 dB) [90, 91] with a trend toward increasing asymmetry among higher frequencies or with increasing levels of hearing loss [92]. There is a margin of error for audiometric testing of ± 9.6-14.2 dB for single frequencies, with the largest range reported at 4 kH [93], which needs to be considered when documenting asymmetric hearing loss. Furthermore, these small differences are based on mean hearing thresholds of group data, which probably underestimates the asymmetric effect of noise exposure at the individual level.

It is worth considering some study findings in more detail. In a study of 208 patients, Fernandes et al. identified asymmetrical hearing loss in 22.6%, of which 6.4% had a definite history of asymmetrical noise exposure and in whom 60% had greater hearing loss in the left ear [94]. Chung et al. found a prevalence of asymmetrical hearing loss in 4.7% among 1461 patients with noise-induced hearing loss and the left ear was affected more in 82.6% [95]. Alberti et al. found a 15% prevalence of asymmetrical hearing loss in 1873 patients referred for compensation assessment, and concluded that 36% of patients with asymmetrical hearing loss were attributable to noise exposure, due to a definitive pattern of hearing loss and a history of noise exposure [1]. In truck drivers, asymmetrical hearing loss has been attributed to noise and air rushing from the opened window [96]. Chung et al. showed that intensity of noise exposure from sawing wooden blocks into shingles was comparable between both ears, but their data also showed a small but significant asymmetric hearing loss, worse on the left side, that correlated with age and lifetime noise exposure when compared to the industrial population [97]. In addition, a significant asymmetrical hearing loss of up to a >20 dB difference was found in different studies of populations evaluating symmetrical noise exposure [98–100]. Major limitations of these studies include reliance on self-reported historical exposures to noise, limited data on the extent of noise exposure, inconsistent criteria for the diagnosis of asymmetrical hearing loss, small sample size, lack of a control group without noise exposure, and lack of direct measures of the physiology of the ear over time.

Studies over the last two decades using industrial or continuous noise exposures have found that noise affects the left ear more than the right ear [101, 102]. A similar observation was reported for exposure to impulse sounds, such as gunshots [63, 103]. Interestingly, other studies have found no significant correlation between usage of firearms and asymmetry of hearing loss, although the left ear was exposed to more of the noise of the gun blast [101, 104]. Tinnitus was also reported to be more frequent in the left ear than the right ear [105, 106]. The lateral difference with hearing in the left ear being worse than the right increases with frequency and reaches a peak at 3–6 kHz. In fact, correlation studies looking at 2 kHz asymmetry suggest that as more frequencies are considered, more patients with asymmetrical hearing loss are likely to be found, and the degree of asymmetry can be more precisely delineated [95]. Chung et al. reported the left ear to be most susceptible to noise at 2 kHz, which may account for a small but significant interaural threshold difference [95, 97]. Pirila et al. reported damage to the left ear to be more prominent in men than in women [107, 108], whereas Nageris et al. found no such difference. With regard to age, Pirila et al. noted that in children aged 5 to 10 years, there was no left or right predominance in hearing loss [109]. They postulated that the difference developed later in life and was at the level of the inner ear. Other groups also noted no effect of left- or right-handedness on hearing loss asymmetry [63, 103].

### Pathophysiology of asymmetric NIHL

Asymmetry in NIHL could theoretically be caused by ambient exogenous noise-exposure factors or by endogenous or anatomical factors. For instance, differentially shielding the right ear from noise or acoustic-energy emitting sources, termed the head shadow effect, may play a role in asymmetric hearing loss [110]. Significant asymmetry will theoretically occur if the noise source is closer in proximity to one side than the other, for instance in workers using hand-held tools predominantly in one hand [111] or in military personnel with weapon noise exposure [103]. The handedness of the subject should thus be of relevance. Since most individuals are right-handed, the muzzle blast from a shotgun reaches the left ear at a higher level than the somewhat shielded right ear. However, studies assessing the impact of handedness on hearing loss showed no correlation between the ear with the asymmetry and the individual’s handedness [63, 103]. Several confounding factors are of relevance though. Some left-handed subjects have always fired right-handed or have changed from left to right during their careers; some rifles in use are now right-hand fire only. For most other weapons, the firing position is fixed and therefore the amount of noise exposure to each ear is determined by the head position relative to the weapon [92]. Other factors to be taken into account include the unilateral use of ear defenders, such as in radio operators where the possible noise hazard or the protective effect can come from use of the headset [112–114]. In industry, most workers also tend to look over their right shoulder when they operate heavy equipment, and thus their left ear is more exposed to the noise generated by the machine’s engine [115]. However, the persistent inferiority of the left ear in most of the studied noise-exposed and normal hearing populations suggests that the head shadow effect cannot be the only factor leading to asymmetric NIHL.

Alternatively, the left ear may somehow be more susceptible to NIHL than the right ear, regardless of exogenous noise exposure factors, and this translates into an asymmetric pattern of hearing loss in both noise-exposure and general non-noise exposure populations [89, 103, 110]. The notion that the left ear is the “weaker” ear in most instances is also supported by the fact that tinnitus in the left ear tends to be more magnified than the right ear [105, 106]. Individual differences in ear anatomy and physiology, or differences in biological recovery from noise exposure may be responsible. Johnson and Sherman examined the acoustic reflex mechanism given its role as a major protective vehicle against acoustic trauma [116]. In children aged 6 to 12 years with normal hearing, it was discovered that the acoustic reflex threshold in the right ear was 3 to 7 dB lower than the left ear [116]. However, this finding was not able to be replicated in adults [95]. Arguably, the protective effect of the stapedial reflex is most efficient in the low frequency range, and may not be as important at frequencies higher than 2 kHz [117, 118]. In short, the protective role of the efferent pathways to cochlea and the possible left-right asymmetries in this system need further research [119, 120].

### Clinical relevance of asymmetric NIHL

Unilateral or asymmetrical sensorineural hearing loss is important to discern, as it can be a hallmark symptom/sign of a retrocochlear lesion (i.e. vestibular schwannoma), and in such cases further investigation is required (i.e. MRI scan) unless there is a known reason for the asymmetry [121]. Hence, recognition of asymmetrical hearing due to noise exposure through careful history taking may optimize more appropriate cost-effective investigation of patients.

Conventional teaching suggests that a claimant for compensation who has occupational hearing loss with asymmetrical hearing thresholds is unlikely to have a noise-induced hearing loss in the worse ear, and like any other patient, should be investigated for the ‘other’ cause of the asymmetry. However, given the multitude of recent evidence in the literature, if the asymmetry under question cannot be explained by causes other than noise, and the MRI scan does not reveal another cause, then the decision given should be in favour of the worker, on the basis of benefit of doubt [94] as the asymmetry may represent a lateral difference in susceptibility to noise damage.

## Beyond hearing loss: associated symptomatology

### NIHL and tinnitus

The prevalence of tinnitus among noise-exposed workers is much higher (24%) than the overall population (14%) [122], and is exponentially higher in those in the military, up to 80% [123]. Although the majority of individuals with NIHL present with bilateral tinnitus, unilateral tinnitus is reported as well, with a prevalence of up to 47% [124–126]. Tinnitus is more prevalent on the left side [124, 125] consistent with the asymmetry documented in NIHL. The severity of the tinnitus may be associated with the degree of NIHL [126, 127]. The impact of tinnitus has been demonstrated: apart from tinnitus being associated with other comorbidities, such as anxiety, depression and sleep disorders [128], noise-induced tinnitus negatively effects the quality of life in workers [129] and for military personnel, tinnitus can be distracting during a military operation [123].

### NIHL and vestibular dysfunction

There is increasing evidence for noise-induced vestibular deficiency, through a mechanism of noise-induced damage to the sacculocolic reflex pathway and/or damage to the vestibular hair cell cilia [62, 130]. This is supported by multiple studies in human and animals.

In humans, several studies, with relatively small sample sizes (*n* = 20-30), showed that abnormal (reduced, delayed or absent responses) cervical vestibular evoked myogenic potentials (VEMPs) and ocular VEMPs are associated with chronic or acute acoustic trauma [62, 131–133]. This supports the hypothesis that noise causes functional damage to the otolithic organs either directly or indirectly. Also, an association was found between cervical VEMPs and hearing outcome after acute acoustic trauma, therefore it was concluded that abnormal VEMPs might indicate more severe trauma and as a result poorer hearing recovery [62].

Apart from the otolithic organs, noise induced trauma has been shown to cause substantial stereocilia bundle loss and reduction in baseline firing rates of (horizontal and superior) semicircular canals in animal studies [130, 134]. A study of 258 military males identified a strong correlation between vestibular symptoms and abnormal findings on electronystagmography (ENG) testing; the presence of spontaneous, gaze-evoked or positional nystagmus and reduced caloric responses in the worst hearing ear was demonstrated, with significantly more abnormal results of all ENG tests in the asymmetrical NIHL group compared to the group with symmetrical NIHL [135]. In these patients, reduced caloric responses were measured in the worst hearing ear, with the left ear being more often affected, suggesting that acoustic trauma can cause asymmetric noise-induced vestibular loss. Whether or not individuals with symmetrical hearing loss also have bilateral symmetrical vestibular hypofunction cannot be gleaned from the data as absolute values were not reported. Data from this study not only supports the hypothesis that acoustic trauma can cause damage to the (horizontal) semicircular canals, but also shows evidence for asymmetrical trauma after noise exposure, in line with previously discussed evidence for asymmetric induced hearing loss (see paragraph “Asymmetric NIHL”).

In animals, noise exposure resulted in a reduction in stereocilia bundle density in vestibular end organs as well as a reduction in regular vestibular afferent baseline firing rates of the otolithic organs and the anterior semicircular canal [130]. As a normal vestbulo-ocular reflex was measured, it was concluded that noise-induced vestibular damage can be present even in the setting of normal vestibular tests; comparable to “hidden hearing loss”, this might indicate that noise exposure can also cause “hidden vestibular loss” that cannot be identified due to limitations in current techniques for vestibular assessment. This might explain why normal or marginally abnormal vestibular function tests can be seen in noise-exposed individuals [136, 137]. Although the impact of noise-induced vestibular loss is unknown, it may explain why individuals with NIHL may present with balance disorders and dizziness [135, 138] and therefore needs to be considered when evaluating the impact of noise-induced trauma.

### The socio-economic impact of NIHL

The United States Government Accountability Office report on noise (2011) indicated that hearing loss was the most prevalent occupational health disability in the Department of Defense (DoD) [123]. The DoD civilian worker compensation costs were approximately $56 million in fiscal year 2003, and Veterans Affairs compensation costs were approximately $1.102 billion in fiscal year 2005 with hearing loss as second most common type of disability [12]. The World Health Organization reported that hearing loss is in the top three common health conditions related to disability in the world as of 2017 [139, 140].

The consequences of occupational NIHL to the individual, although not life-threatening, can be dire. Hearing loss limits an individual’s ability to communicate with the surrounding world, which can lead to increased social stress, depression, embarrassment, poor self-esteem, and relationship difficulties [59]. Social handicap resulting from communication difficulties is exacerbated in difficult listening situations, such as environments with excessive background noise. In addition, longitudinal studies have demonstrated an association between hearing loss and declines in cognition, memory, and attention signifying the importance of prevention and treatment of hearing loss [141, 142].

Occupational NIHL has been associated with an increased risk for work-related injuries. For each dB of hearing loss, a statistically significant risk increase was observed for work-related injuries leading to admission to hospital [143]. Individuals with asymmetrical NIHL may experience decreased ability to localize sounds, which is critical in certain groups of workers like firefighters and other public safety workers, and can be a career-ending disability that has public safety implications as well [144].

## Non-pharmaceutical interventions

### Education, regulations, legislation and workplace noise policy

Prevention remains the best option for limiting the effects of acoustic trauma. Hearing conservation programs in elementary school children are potentially effective to increase the knowledge about the hazards of noise exposure early in life and this may result in behavioral changes towards noise reduction and ear protection [145]. For industrial noise, elimination or reduction of noise through engineering or administrative controls is the best line of defense. Legislation on occupational noise exposure help to regulate noise exposure and result in noise reduction and/or noise reducing technical improvements to protect employees [146].

The risk of NIHL can be minimized if noise is reduced to below 80 dB(A) (weighted decibel relative to human ear) [147]. For higher levels of noise, regulations are necessary as the extent of biological damage correlates directly to the total sound energy level, a function of sound pressure (decibels) and the duration of exposure (time) [9]. Hearing loss prevention programs establish permissible exposure limits with an exchange rate. The exchange rate defines the number of decibels by which the sound pressure level may be decreased or increased for a doubling or halving of the duration of exposure. This principle is reflected in occupational exposure limits for workplace noise with maximum daily exposure limits halved for every 3–5 dB increase in noise intensity. For instance, assuming an exchange rate of 3 dB, 4 h of exposure at 88 dB(A) is as equally hazardous as 8 h at 85 dB(A).

A recent Cochrane review concluded that in order to prevent occupational hearing loss, better implementation of legislation and better prevention programs are necessary [148]. Regulations vary widely among different countries and one third of countries in the world still do not have regulations or legislation regarding permissible noise levels and exchange rates [149]. Most North and South American countries have the permissible exposure limit (PEL) of 85 dB(A) for an 8 h work day [149]. In some countries (and some provinces in Canada), the PEL is up to 90 dB(A). As TTSs are higher when workers are exposed to 90 dB(A) as compared to 85 dB(A), a standardized reduction of the PEL to 85 dB(A) should be established in order to reduce the prevalence of NIHL [150]. There is also no international consensus regarding the exchange rate, which varies between countries from 3 dB to 5 dB [149]. There is evidence, however, that 3 dB overestimates the risk of NIHL and that 5 dB is a better fit [151]. For impulse noise, there is most often a limit of peak sound pressure level of 140 dB [152].

## Hearing protection

Hearing protection offers a secondary level of protection. However, evidence for effective hearing loss prevention programs (using personal hearing protection) is limited. The most effective hearing protection, including earmuffs and earplugs, can reduce loud noise trauma, but compliance may be limited due to the impact on one’s ability to communicate when they are worn and/or discomfort related to their use [153, 154]. To promote the use of hearing protection, different interventional strategies may be beneficial, such as providing general information to motivate workers to use hearing protection or more personalized programmes that provide specific information regarding the risks to the individual worker [155]. There is a trend towards improved hearing protective device use when a tailored strategy is used that is either situation specific or individual specific, compared to a non-tailored strategy [156]. Hearing protection with lower attenuation but higher comfort is more efficient than protection with higher attenuation but lower comfort due to compliance issues [157, 158]. Custom earplugs have a more consistent attenuation than non-custom earplugs, and user training can improve consistency [159]. Individual fit-testing, which measures the effectiveness of hearing protection devices specifically for each individual, can be invaluable, particularly with earplugs since they are generally less consistent in noise reduction than ear muffs [160]. For earmuffs, new materials and design can potentially improve comfort and hearing protection. A recent published study using 3D printed earmuffs showed that the use of light materials like acrylonitrile butadiene styrene/clay nanocomposites can reduce the weight of earmuffs without reducing the attenuation performance [161]; such technological advancements have the potential to increase comfort and improve compliance.

## Pharmacological treatments

### Anti-inflammatory effects of corticosteroids to reduce noise induced trauma

Different types of pharmaceutical agents have been shown to reduce the risk of hearing loss secondary to acoustic trauma. Steroids, specifically intratympanic dexamethasone, may have a therapeutic beneficial effect on NIHL when given before [162] or after [163] acoustic trauma in animals. Although an effect is shown in a wide range of dosages, higher dosages appear to be associated with better hearing preservation [162].

Different routes of delivery have been investigated in animals, including intratympanic, intraperitoneal and direct administration into the scala tympani, and all have demonstrated protective effects as evidenced by preserved hearing (15–20 dB lower hearing thresholds on auditory brainstem response (ABR) measurement and preserved cochlear architecture [163, 164]. Each route of delivery may protect hearing at a different level; intratympanic administration appears to be more protective for the efferent terminal outer hair cells synapses, whereas intraperitoneal injections are more protective for the organ of Corti and stria vascularis architecture [163]. Accordingly, there appears to be a synergistic benefit from the administration by both routes when treating NIHL [165]. In human studies, it has been shown that after acoustic trauma, the administration of systemic with intratympanic steroid treatment results in better hearing outcomes than with systemic steroids alone [165, 166]. Although there is some evidence for a protective effect of steroids in acute acoustic trauma, clearly it is not a long-term option for chronic occupational noise exposure considering the negative side effects of systemic long-term steroid usage.

### Antioxidants to reduce oxidative stress

Antioxidants may be a safer alternative to steroids given a more favourable side effect profile. Free oxygen radicals and oxidative stress are important in the pathogenesis of the NIHL, and therefore antioxidants could theoretically constitute an effective treatment.

N-acetylcysteine (NAC) has been reported to reduce the ototoxic effects of noise exposure in animal models [167–171]. In humans, however, the data is limited [172–174]. Doosti et al. evaluated TTS in 48 textile workers and showed that daily oral administration of NAC (1200 mg/day) during continuous noise exposure prevented the occurrence of a TTS after 14 days of treatment, whereas the untreated group showed a TTS of approximately 1.5–3 dB [172]. Lin et al. also found a significant improvement in TTS after NAC (1200 mg/day for 14 days). However, the mean difference in TTS in the placebo-treated group versus NAC-treated group was only 0.3 dB [175]. Kramer et al. did not find a significant protective effect of NAC when using a single lower dose (900 mg PO) administered before noise exposure [173]. A more recent randomized, double-blinded, placebo-controlled trial among a larger military group (n = 566), found a 6–7% reduction in hearing threshold shift rate, with a total daily dose of 2700 mg of NAC after noise exposure for 16 days during weapon training, but this was only statistically significant when handedness was taken into account (i.e. evaluating the effect on the right ear only in right handed participants). In summary, there is potentially a small benefit of NAC in reducing the rate of threshold shift in a noise-exposed population [176].

Other antioxidants that can potentially play a protective role against noise-induced cochlear trauma include ginseng [172], co-enzyme Q10 [177], as well as several vitamins, such as vitamin A [178], vitamin C [179, 180], vitamin E [181, 182], and vitamin B12 [183]. Studies in animals showed a protective benefit from combination antioxidant treatment, such as magnesium and vitamin A, C, and E [184], possibly due to synergistic effects [185–187], These studies were mainly performed in animals or in small groups of humans and the results should be considered preliminary. The efficacy of combining treatments in humans is still unknown.

### Neurotrophins for recovery of ribbon synapses

There is evidence in animals that neurotrophins can offer protective effects against noise trauma [188–191]. Neurotrophin-3 (NT3) and brain derived neurotrophic factor (BDNF) are important for formation and maintenance of hair cell ribbon synapses in the cochlea, as well as in the vestibular epithelia [190]. NT3, derived from supporting cells, promotes the recovery of the number of ribbon synapses as well as their function after noise-induced trauma [189, 190]. A dose-dependent effect was found of glial cell-derived neurotrophic factor (GDNF) on sensory cell preservation as well as ABR confirmed hearing threshold, after chronic application of GDNF (10 and 100 ng/ml) through a cochleostomy in the scala tympani via a micro-osmotic pump. However, this effect was small and appears to be associated with some toxicity at a higher concentration (1 μg/ml) [188]. Even a single application of NT3 and BDNF on the round window, immediately after noise trauma, can potentially reduce the synaptopathy (indicated by increased number of presynaptic ribbons, postsynaptic glutamate receptors, and co-localized ribbons) and recover hearing [191]. Another approach is transplantation of neurotrophin-secreting olfactory stem cells into the cochlea, which also caused restoration of noise-induced hearing loss [192]. Although these results are promising, long-term effects are still unknown and no studies in humans have been performed to date.

### Other pharmaceutical agents

Other pharmaceutical agents with possible protective NIHL effects include magnesium and statins. A human study [193] as well as research on animal models [194, 195] have shown that acoustic trauma can potentially be minimized by magnesium, as it reduces apoptosis of hair cells by a reduction of calcium flow into the cell, thereby reducing reactive oxygen species formation. A double-blinded, placebo-controlled, crossover trial to assess the effects of prophylactic N-acetylcysteine (600 mg) and magnesium (200 mg) prior to noise exposure is pending [196].

Statins might prevent NIHL by reducing oxidative stress and improving hair cell survival in animals [197, 198]. A significant recovery of TTS (determined by measuring distortion product otoacoustic emissions) was found in rats treated with 5 mg/kg atorvastatin administered daily for 2 weeks prior to 2 h of noise exposure [199].

## Surgical treatment

### Cochlear implantation

Due to the severity of the hearing loss and/or the poor speech recognition due to synaptopathy, some individuals with NIHL might eventually become candidates for cochlear implantation (CI) either with full electrical or with electro-acoustic stimulation (EAS). Studies have reported NIHL as the etiology of deafness in implanted individuals, with a prevalence ranging from 2% (CI) to 20% (CI with EAS) [200, 201]. This may underestimate the true prevalence, considering the high percentage of unknown etiologies approximating 40–50% of CI recipients [200]. Currently we can only speculate on the extent to which the SNHL in these implanted individuals can be attributed to noise exposure or due to a combination of other underlying predisposing factors.

## Conclusion

The impact of noise-induced hearing loss is more widespread than has previously been recognized. Apart from a wide range of hearing frequencies that can be adversely affected by noise exposure, there is increasing evidence that noise-induced synaptopathy causes reduced speech perception in noise, even when pure tone thresholds are still preserved (“hidden hearing loss”). Evidence in the current literature further supports the notion that noise exposure can result in an asymmetric pattern of hearing loss due to unique differences in susceptibility to noise damage within individuals, increase frequency of tinnitus as well as vestibular dysfunction. The left ear (hearing and balance) is more adversely affected by noise, even in the presence of symmetric noise exposure. Future studies should focus on underlying mechanisms that lead to the susceptibility of left-right asymmetry, and to understand the protective role of the efferent pathways to the cochlea as demonstrated in gender differences. Primary prevention with a focus on regulations, legislation and education in schools, in combination with proper hearing protection are important first lines of defense. Further human studies are needed to address the effectiveness of pharmaco-therapeutic options to prevent or mitigate noise-induced trauma.

## Abbreviations

ABR: Auditory brainstem response
dB(A): A-weighted decibel
dB: Decibel
ENG: Electronystagmography
kHz: Kilohertz
NAC: N-acetylcysteine
NIHL: Noise-induced hearing loss
OAEs: Otoacoustic emissions
PEL: Permissible exposure limit
PTS: Permanent threshold shift
ROS: Reactive oxygen species
SNHL: Sensorineural hearing loss
SPL: Sound pressure level
TTS: Transient threshold shift
VEMPs: Vestibular evoked myogenic potentials
